# Pathogenicity of Pandemic H1N1 Influenza A Virus in Immunocompromised Cynomolgus Macaques

**DOI:** 10.1371/journal.pone.0075910

**Published:** 2013-09-23

**Authors:** Van Loi Pham, Misako Nakayama, Yasushi Itoh, Hirohito Ishigaki, Mitsutaka Kitano, Masahiko Arikata, Hideaki Ishida, Naoko Kitagawa, Shintaro Shichinohe, Masatoshi Okamatsu, Yoshihiro Sakoda, Hideaki Tsuchiya, Shinichiro Nakamura, Hiroshi Kida, Kazumasa Ogasawara

**Affiliations:** 1 Division of Pathology and Disease Regulation, Department of Pathology, Shiga University of Medical Science, Otsu, Japan; 2 Department of Otorhinolaryngology-Head and Neck Surgery, Shiga University of Medical Science, Otsu, Japan; 3 Research Center for Animal Life Science, Shiga University of Medical Science, Otsu, Japan; 4 Laboratory of Microbiology, Department of Disease Control, Graduate School of Veterinary Medicine, Hokkaido University, Sapporo, Japan; 5 Research Center for Zoonosis Control, Hokkaido University, Sapporo, Japan; German Primate Center, Germany

## Abstract

Pandemic (H1N1) 2009 influenza virus spread throughout the world since most people did not have immunity against the virus. In the post pandemic phase when many humans might possess immunity against the pandemic virus, one of the concerns is infection in immunocompromised people. Therefore, we used an immunosuppressed macaque model to examine pathogenicity of the pandemic (H1N1) 2009 virus under an immunocompromised condition. The virus in nasal samples of immunosuppressed macaques infected with the pandemic (H1N1) 2009 virus was detected longer after infection than was the virus in nasal samples of immunocompetent macaques. As expected, not only virus amounts but also virus propagation sites in the immunosuppressed macaques were larger than those in lungs of the immunocompetent macaques when they were infected with the pandemic virus. Immunosuppressed macaques possessed low levels of immune cells producing cytokines and chemokines, but levels of inflammatory cytokines/chemokine interleukin (IL)-6, IL-18, and monocyte chemotactic protein (MCP)-1 in lungs of the immunosuppressed macaques were higher than those in lungs of the immunocompetent macaques, though the differences were not statistically significant. Therefore, under an immunosuppressive condition, the pandemic influenza (H1N1) 2009 virus might cause more severe morbidity with high cytokine/chemokine production by the host innate immune system than that seen in macaques under the immunocompetent condition.

## Introduction

A pandemic H1N1 influenza virus emerged and spread throughout the world in 2009 [1]. Most of the people except for those born before 1918 might have been susceptible to the pandemic (H1N1) 2009 virus because of the lack of a neutralization antibody against the virus [2]. The pandemic (H1N1) 2009 virus also caused severe pneumonia since it was shown that the pandemic virus propagated more vigorously than did a seasonal influenza virus (Russian flu virus) in the lungs of animal models and human patients [2-6]. After the World Health Organization declared a postpandemic phase in August 2010 (http://www.who.int/mediacentre/news/statements/2010/h1n1_vpc_20100810/en/index.html) [7], the pandemic (H1N1) 2009 influenza virus has been recognized as a seasonal influenza virus. Although several variations in a hemagglutinin (HA) protein in the pandemic (H1N1) 2009 influenza virus and its descendent virus have been reported [8,9], no remarkable variations that required change of a vaccine strain have been reported as of the 2012-2013 season [10,11] (http://www.who.int/influenza/vaccines/virus/candidates_reagents/summary_a_h1n1_cvv_20120308.pdf). Therefore, antigenicity of the pandemic (H1N1) 2009 virus seems to have been maintained even after it became a seasonal influenza virus. Since many people might possess immunity against the pandemic strain due to infection and vaccination after 2009, we need to pay further attention to immunocompromised people as well as elderly and young people who have low levels of immune responses against the virus.

Non-human primates have been used to extrapolate pathogenicity of various pathogens in humans. We reported that the pandemic (H1N1) 2009 virus propagated in the lungs of immunologically naïve macaques more vigorously than did the seasonal Russian flu virus [2]. Indeed, viral pneumonia caused by the pandemic (H1N1) 2009 virus was reported in humans during the pandemic. Immunosuppression altered morbidity induced by pathogens in macaques. For example, in simian immunodeficiency virus (SIV) infection models, microsporidia infection might contribute to morbidity [12] as reported in humans [13]. Irradiation, another method to induce immunosuppression, made rhesus macaques susceptible to methicillin-resistant *Staphylococcus* [14] as was reported in humans [15]. After transplantation and immunosuppression, simian parvovirus infection caused severe anemia in cynomolgus monkeys [16] as reported in humans, in which parvovirus infection usually caused erythema infectiosum (fifth disease) in childhood [17,18]. In addition, immunosuppression allowed pathogens to spread systemically. Neurotropic SV40 spread through the blood to other organs in SIV-infected macaques [19] as was human JC virus detected in blood and urine samples of human immunodeficiency virus (HIV)-infected patients [20]. Treatment of macaques with anti-thymocyte globulin, cyclophosphamide, and cortisone acetate caused a systemic active cytomegalovirus (CMV) infection [21]. Therefore, immunosuppression made pathogens proliferate and spread to unusual propagation sites, resulting in increase of pathogenicity in macaques as well as humans.

To reveal the pathogenicity of influenza virus in immunocompromised hosts compared to that in immunocompetent hosts, we used an immunosuppressed macaque model and examined symptoms and virus propagation. We also compared pathogenicity of pandemic (H1N1) 2009 influenza virus to that of the seasonal H1N1 influenza virus isolated in 2007 (Russian flu virus) in immunosuppressed macaques. Our immunosuppressive protocol decreased the concentration of white blood cells, especially lymphocytes in peripheral blood, and inhibited interleukin-2 (IL-2) responses of lymphocytes. Under the immunosuppressed condition, propagation of pandemic (H1N1) 2009 and Russian flu viruses in the respiratory tract was prolonged compared to that under the immunocompetent condition. Furthermore, Russian flu virus was detected in the lungs of immunosuppressed macaques but not in the lungs of immunocompetent macaques. Even under the immunocompromised condition, levels of cytokine production 7 days after infection in the lungs of immunosuppressed macaques were higher than those in the lungs of immunocompetent macaques. These results suggest that immunosuppressive treatment to inhibit inflammation might not necessarily ameliorate hypercytokinemia and symptoms in influenza virus infection.

## Results

### Immunosuppression by cyclophosphamide and cyclosporine A in cynomolgus macaques

**Figure 1:**
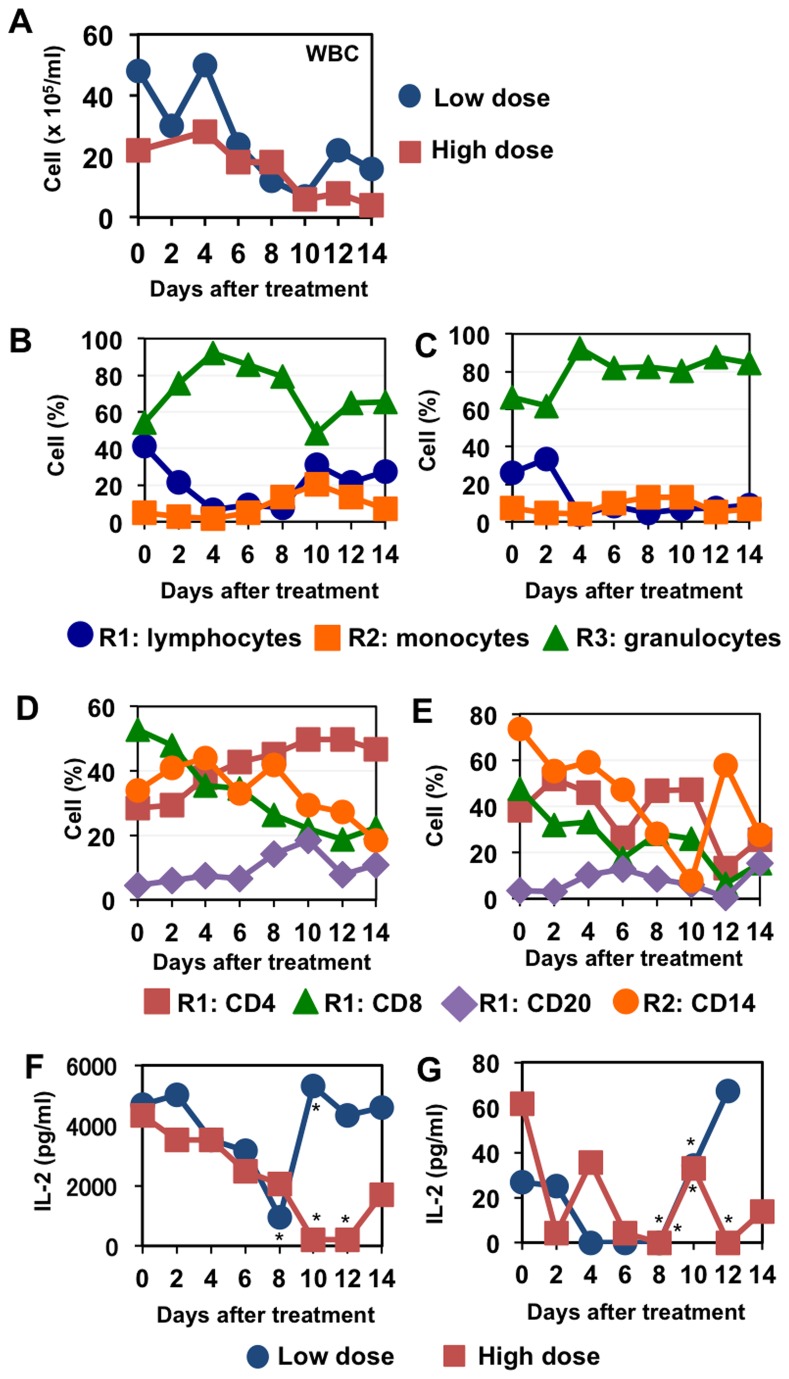
Blood cell population and IL-2 production after immunosuppression. Peripheral blood was collected on the indicated days after the first administration of CP and CA. (A) The number of white blood cells was counted using a microscope and a hemocytometer. (B, C) The percentage of white blood cell population was calculated on the basis of forward and side scatter scales (FSC and SSC) in flow cytometric analysis. Representative profiles of flow cytometric results are shown in Figure S1. Lymphocytes, monocytes, and granulocytes were determined as populations in R1 (low FSC/low SSC), R2 (high FSC/low SSC), and R3 (high FSC/high SSC), respectively. (D, E) The percentages of CD4^+^, CD8^+^, CD20^+^, and CD14^+^ cells were determined with a flow cytometer. The percentages of CD4^+^, CD8^+^, and CD20^+^ cells are indicated as the percentages in R1. The percentages of CD14^+^ cells are indicated as the percentages in R2. B, D: a monkey treated with low CP and CA, C, E: a monkey treated with high CP and CA. (F, G) Peripheral blood cells were culture with PMA+ionomycin (F) or anti-CD3 + anti-CD28 + anti-CD49d antibody (G) for 24 h. IL-2 in supernatants was measured with ELISA. Results are shown as averages of triplicate cultures except asterisked points in which results were calculated from less than three wells since sufficient cells to make triplicate cultures were not collected.

To establish a protocol for suppressing the immune system in cynomolgus macaques, we firstly counted white blood cell numbers in blood as a biomarker for effective immunosuppression (i.e., leukocytopenia). According to previous studies [22-25], we compared two regimens, high-dose and low-dose cyclophosphamide (CP) treatment with cyclosporine A (CA) (Table 1). The concentrations of CA in plasma of macaques treated with high-dose and low-dose CP on day 6 were 65 and 23 ng/ml, respectively. In the blood of a macaque treated with the low dose regimen, the white blood cell count was decreased 7 days after the beginning of suppressive treatment and it partially recovered on day 12 (5 days after the end of treatment), whereas in the high dose regimen, decrease in white blood cell count continued until day 14 (7 days after the end of treatment) (Figure 1A).

**Table 1:** Immunosuppression protocol before influenza virus infection.

**Days after suppression^1^**	**Low dose regimen**		**High dose regimen**	
	**CP^2^ (mg/kg**)	**CA^2^ (mg/kg**)	**CP (mg/kg**)	**CA (mg/kg**)
0	20	50	40	50
1	-^2^	50	-	50
2	20	50	40	50
3	-	50	-	50
4	20	50	40	50
5	-	50	-	50
6	20	50	40	50

1 Days after the beginning of suppression used in Figure 1.

2 CP: cyclophosphamide, CA: cyclosporine A. - without administration.

Next, we examined the population of white blood cells using a flow cytometer. Lymphocytes (R1), monocytes (R2), and granulocytes (R3) were identified on the basis of forward and side scatters (Figure 1B, C, Figure S1). In the blood of the macaque treated with the low dose regimen, the percentage of lymphocytes (R1), especially CD8^+^ cells, was decreased instead of increase of granulocyte ratios and the percentage of lymphocytes was recovered on day 10 (3 days after the end of treatment) (Figure 1B, D, Figure S1A). In the high dose regimen, the percentage of lymphocytes (R1), especially CD8^+^ cells, and the percentage of CD14^+^ cells in monocyte population (R2) were decreased on day 6 (Figure 1C, E, Figure S1B). The decrease in number of lymphocytes did not recover until the end of the observation period in the high dose regimen (Figure 1C).

Since CA inhibits the function of calcineurin, resulting in induction of anergy in T lymphocytes [26,27], we measured IL-2 production by mononuclear cells with either phorbol 12-myristate 13-acetate (PMA) + ionomycin (Figure 1F) or anti-CD3 + anti-CD28 + anti-CD49d stimulation (Figure 1G). In both regimens, IL-2 production by stimulated leukocytes and T lymphocytes was severely down-regulated after administration of immunosuppressive agents, though IL-2 secretion recovered after discontinuation of the drugs, especially in the low dose regimen. These results indicated that the high dose regimen was required for suppression and maintenance of immunosuppression during the experiments.

### Influenza Virus Replication in Immunocompromised Macaques

**Figure 2:**
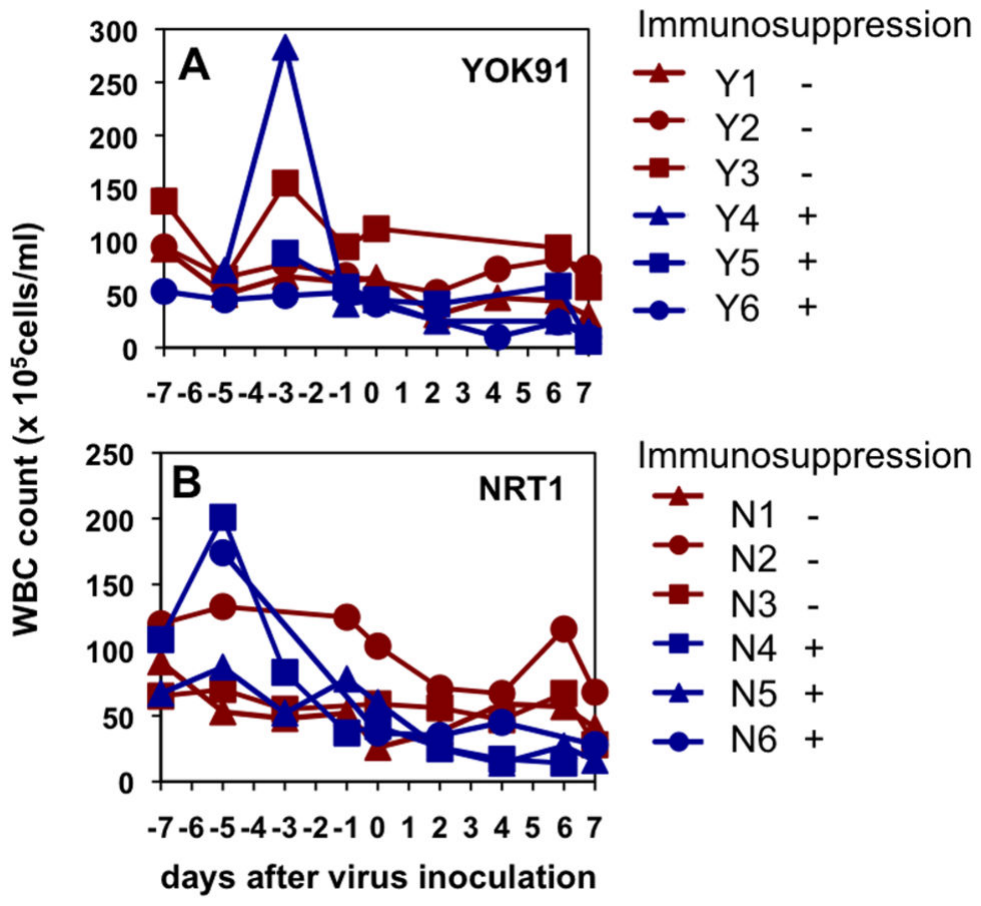
White blood cell counts in peripheral blood after immunosuppression. Total white blood cells were counted using a microscope and a hemocytometer. (A) Macaques infected with seasonal influenza virus A/Yokohama/91/2007 (H1N1) (YOK91) on day 0. (B) Macaques infected with pandemic influenza virus A/Narita/1/2009 (H1N1) (NRT1) on day 0. Red lines: macaques administered saline from day -7 to day 0. Blue lines: macaques administered CP and CA from day -7 to day 0.

To examine the pathogenicity of influenza virus in immunosuppressed macaques, we administered CP and CA in the high dose regimen as determined above (Table 2) and then inoculated the macaques with seasonal Russian influenza virus A/Yokohama/91/2007 (H1N1) (YOK91) or pandemic influenza virus A/Narita/1/2009 (H1N1) (NRT1) into the nostrils, oral cavity, and trachea. All macaques except one (N6), in which the transmitter lost electric power to record, showed higher body temperature than that before infection (Figure S2) even when white blood cell counts were decreased in macaques treated with CP and CA (Figure 2). In macaques inoculated with YOK91 (Y1) and NRT1 (N1 and N2) without immunosuppression, the number of lymphocytes and granulocytes temporally decreased after virus inoculation (Figure S3). These results suggested that high body temperature (fever) after viral infection might not be necessarily dependent on responses of white blood cells.

**Table 2:** Immunosuppression protocol for influenza virus infection.

**Days after infection^1^**	**CP^2^ (mg/kg**)	**CA^2^ (mg/kg**)
-7	40	50
-6	-^2^	50
-5	40	50
-4	-	50
-3	40	50
-2	-	50
-1	40	50
0	-	50

1 Days used in experiments with virus infection.

2 CP: cyclophosphamide, CA: cyclosporine A, - without administration.

**Figure 3:**
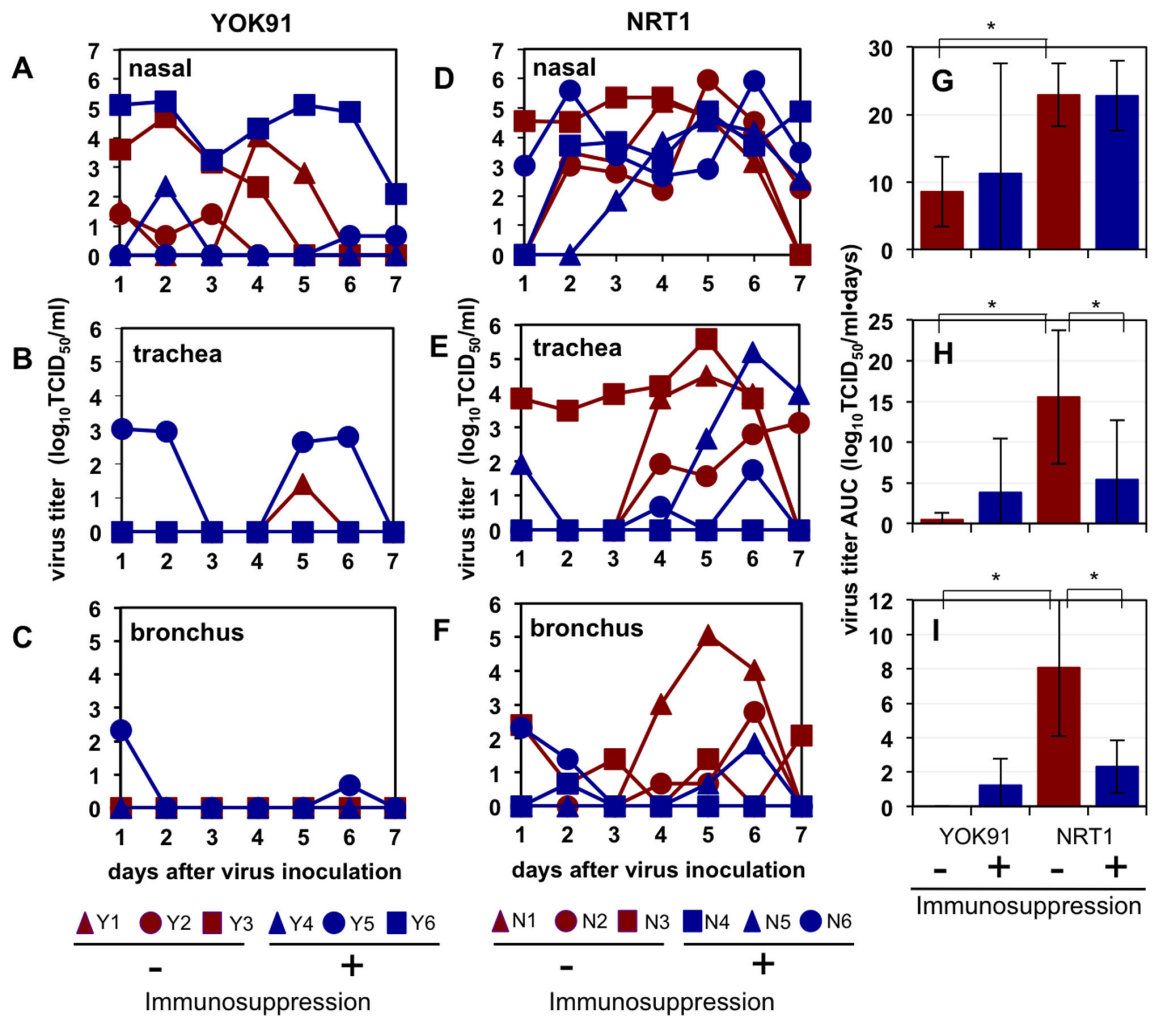
Virus recovery in swab samples from macaques administered immunosuppressive agents. Cynomolgus macaques were administered CP and CA (blue lines) or saline (red lines) from day -7 to day 0 as shown in Table 2 and then inoculated with YOK91 (A-C) or NRT1 (D-F) on day 0. Viral titers in nasal (A, D), tracheal (B, E), and bronchial (C, F) samples were determined using MDCK cells. Virus titers under the detection limit (< 0.67 TCID_50_/ml) are indicated as 0. (G-I) Virus titer areas under the virus titer time curves (virus titer AUC) in nasal (G), tracheal (H), and bronchial (I) samples were calculated on the basis of titers in A-F. Averages and standard deviations of three macaques are shown in each group. Asterisks indicate significant differences (P < 0.05, Student’s t-test).

The virus was detected in nasal samples of all three macaques without immunosuppression challenged with seasonal virus YOK91 until up to day 5 after infection, whereas the virus was detected in nasal samples of two immunosuppressed macaques (Y5, Y6) until day 7 and in tracheal and bronchial samples until day 6 in one of the immunosuppressed macaques (Y5) (Figure 3A-C). A comparison of the immunosufficient and immunosuppressed macaques inoculated with NRT1 showed that virus titers in nasal samples of the immunosuppressed macaques on day 7 were significantly higher than those in nasal samples of immunocompetent macaques (P = 0.046 by Student’s t-test, Figure 3D). These results showed that immunosuppression enhanced propagation of pandemic (H1N1) 2009 virus in nasal swab samples.

We calculated the areas under the virus titer curves (virus titer AUCs) to compare overall virus propagation. Significant differences on average virus titer AUCs in nasal, tracheal, and bronchial samples were observed between the macaques inoculated with YOK91 and those inoculated with NRT1 without immunosuppression (Figure 3G-I, P < 0.05 by Student’s t-test). These results confirmed that the pandemic (H1N1) 2009 virus replicated in macaques more vigorously than did the seasonal Russian flu virus as previously reported [2]. However, virus titer AUCs in the tracheal and bronchial samples of immunosuppressed macaques infected with NRT1 were smaller than those in the samples of immunocompetent macaques (Figure 3H, I).

**Figure 4:**
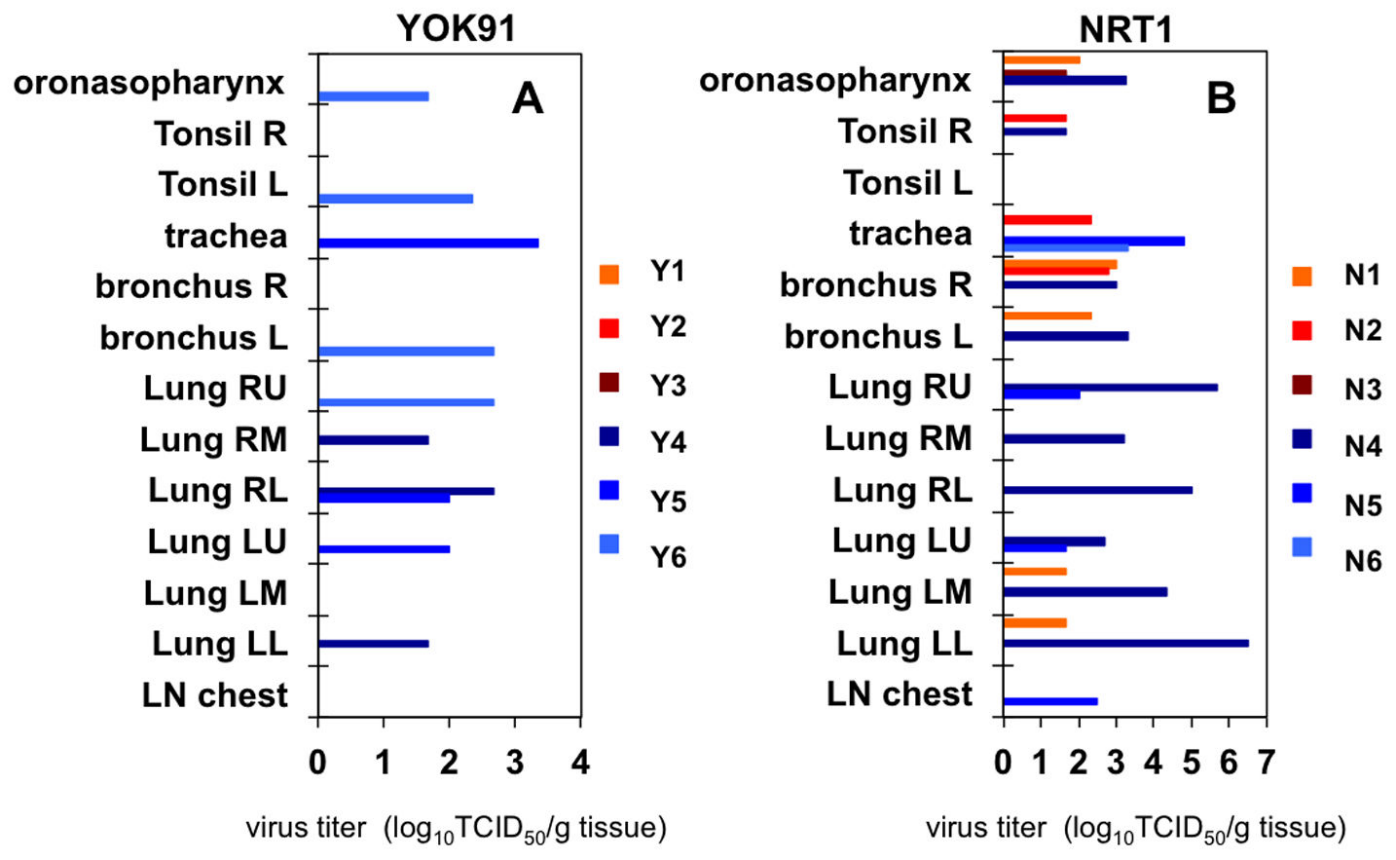
Virus recovery in organ samples from macaques administered immunosuppressive agents. Each macaque was inoculated with saline (Y1-Y3, N1-N3) or immunosuppressive agents (Y4-Y6, N4-N6) as described in the legend for Figure 3. Macaques were inoculated with YOK91 (A, Y1-Y6) or NRT1 (B, N1-N6) on day 0. Tissue samples were collected at autopsy 7 days after virus inoculation. Tissue homogenates were prepared as 10% w/v solution. Virus titers under the detection limit (< 1.67TCID_50_/g tissue) are indicated as 0. R: right, L: left, RU: right upper lobe, RM: right middle lobe, RL: right lower lobe, LU: left upper lobe, LM: left middle lobe, LL: left lower lobe, LN chest: lymph nodes in the mediastinum.

In tissue samples collected at autopsy 7 days after virus inoculation, virus was under the detection limit in all of the immunocompetent macaques infected with YOK91 (Figure 4A). On the other hand, the virus was detected in the upper airway of two immunosuppressed macaques (Y5, Y6) and in one to four lobes of lungs of all immunosuppressed macaques (Y4-Y6). Pandemic virus was detected in oronasopharynx mucosa (N1, N3), a tonsil (N2), trachea (N2), bronchi (N1, N2), and two lobes of the lung (N1) of immunocompetent macaques inoculated with NRT1 (Figure 4B). On the other hand, high titers of pandemic virus were detected in oronasopharynx mucosa (N4), a tonsil (N4), trachea (N5, N6), bilateral bronchi (N4), six lobes of the lung (N4), and two lobes of the lung (N5) in three immunosuppressed macaques. No virus was detected in other organs of immunocompromised macaques infected with YOK91, whereas the pandemic virus was detected in one of two tissue pieces of the cerebellum (2.00 log_10_TCID_50_/g), ileum (1.67 log_10_TCID_50_/g), colon (1.67 log_10_TCID_50_/g), and urinary bladder (1.67 log_10_TCID_50_/g) samples in the N4 macaque, and colon (1.67 log_10_TCID_50_/g) in the N6 macaque when we collected two pieces of tissue samples from each organ. The presence of virus was confirmed by immunohistochemical staining of nucleoprotein (NP) in a small number of type I and type II alveolar epithelial cells in the lung of an immunosuppressed macaque infected with YOK1 (Y4), but not in the lung of an immunocompetent macaque infected with YOK1 (Y3) (Figure S4B, C). NP was detected in bronchiolar epithelial cells in the lung of an immunocompetent macaque infected with NRT1 (Figure S4D), whereas many type II alveolar epithelial cells and a few bronchiolar epithelial cells were positive for NP in the lungs of immunosuppressed macaques infected with NRT1, N4 and N5, respectively (Table 3). The number of cells positive for NP antigen in the immunohistochemically stained sections was compatible with virus titers in lungs, especially in the lung of N4. No NP antigen was detected in the cerebellum, urinary bladder, and colon of N4 probably due to a low antigen concentration (Figure S4F-H). These results indicate higher propagation activity of the pandemic (H1N1) 2009 virus than that of the seasonal Russian flu virus as previously reported [2] and also indicate that immunosuppression enhanced dissemination of both seasonal and pandemic viruses.

**Table 3:** Semiquantitative analysis of virus infected cells with immunohistochemical staining.

Virus	YOK			NRT			NRT		
Suppression	+			-			+		
Animal	Y4	Y5	Y6	N1	N2	N3	N4	N5	N6
Oronasopharynx			-	-		-	-		
Tonsil			-		-		-		
Trachea		-			-		-	-	-
Bronchus			-	-	-		-	-	
Lung	+	-		+*			+/++	+*	
LN chest								-	
Cerebellum							-		
Colon							-		
Urinary bladder							-		

- no virus antigen-positive cells, + some virus antigen-positive cells, ++ many virus antigen-positive cells, +* virus antigen positive in bronchiolar epithelial cells in lungs. Blank: not tested.

### Inflammatory responses in immunosuppressed macaques after influenza virus infection

**Figure 5:**
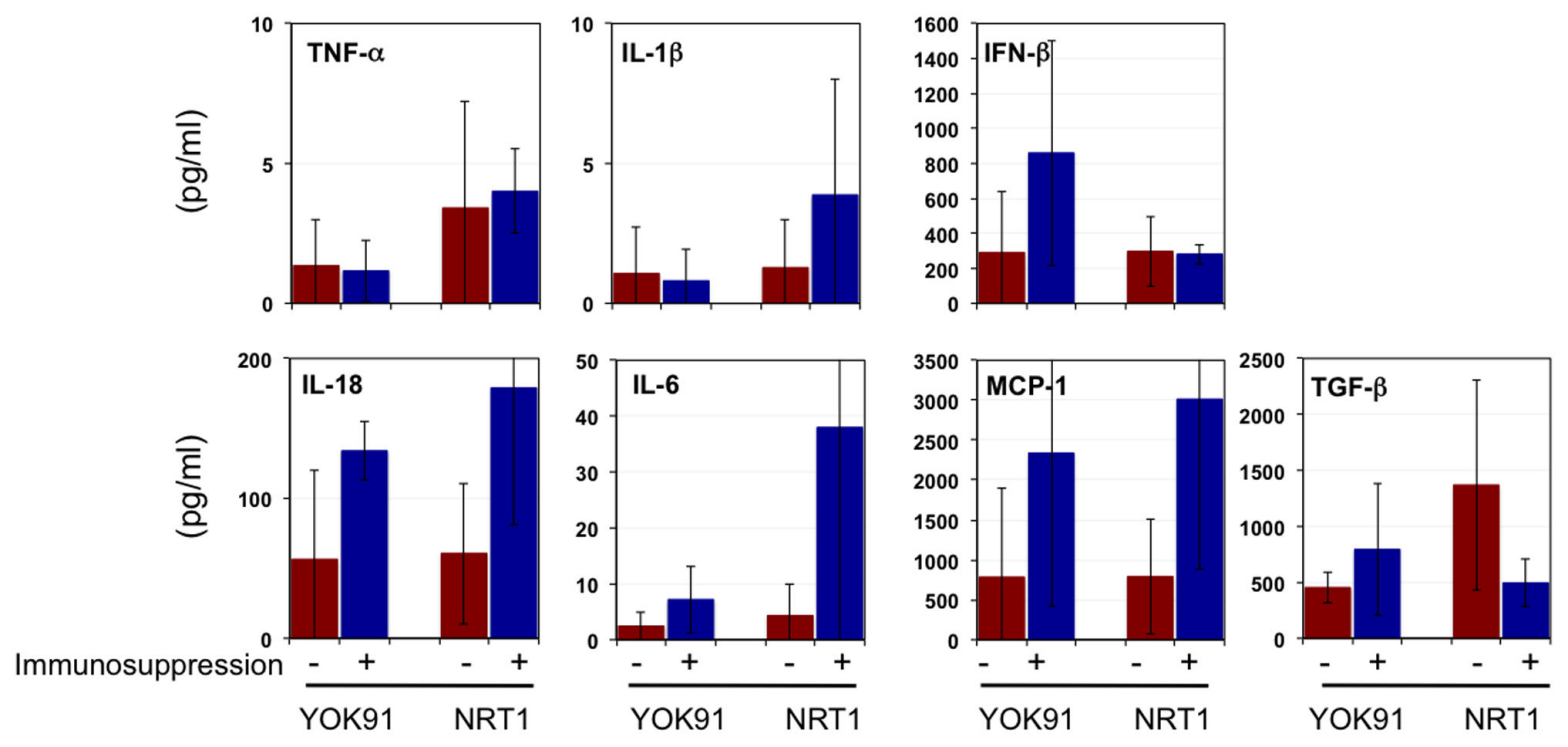
Cytokine production after virus infection in immunosuppressed macaques. Cytokine production in lung tissues was measured. Averages and standard deviations of three macaques are shown. Red bars: without immunosuppression, blue bars: with immunosuppression.

We examined inflammatory responses in the macaques with or without immunosuppression after infection. Cytokine production was examined in lung tissues excised 7 days after virus infection (Figure 5). Levels of tumor necrosis factor (TNF)-α and IL-1β production in the lungs were low in macaques with or without immunosuppression. Although no statistically significant differences were found, concentrations of interferon (IFN)-β, IL-18, and monocyte chemotactic protein (MCP)-1 in lungs of the immunosuppressed macaques infected with YOK91 were higher on average than those in lungs of the immunocompetent macaques infected with YOK91. Average levels of IL-18, IL-6, and MCP-1 production in lungs of the immunosuppressed macaques infected with pandemic NRT1 were higher than those in lungs of the immunocompetent macaques infected with NRT1. On the other hand, transforming growth factor (TGF)-β1 production in lungs of the immunocompetent macaques infected with NRT1 was greater than that in lungs of the immunosuppressed macaques. Therefore, the immunosuppressed macaques showed a tendency to produce relatively high levels of inflammatory cytokines and a chemokine in the lungs after infection with both viruses and to suppress anti-inflammatory TGF-β1 production in the case of pandemic (H1N1) 2009 virus infection, compared to the immunocompetent macaques.

**Figure 6:**
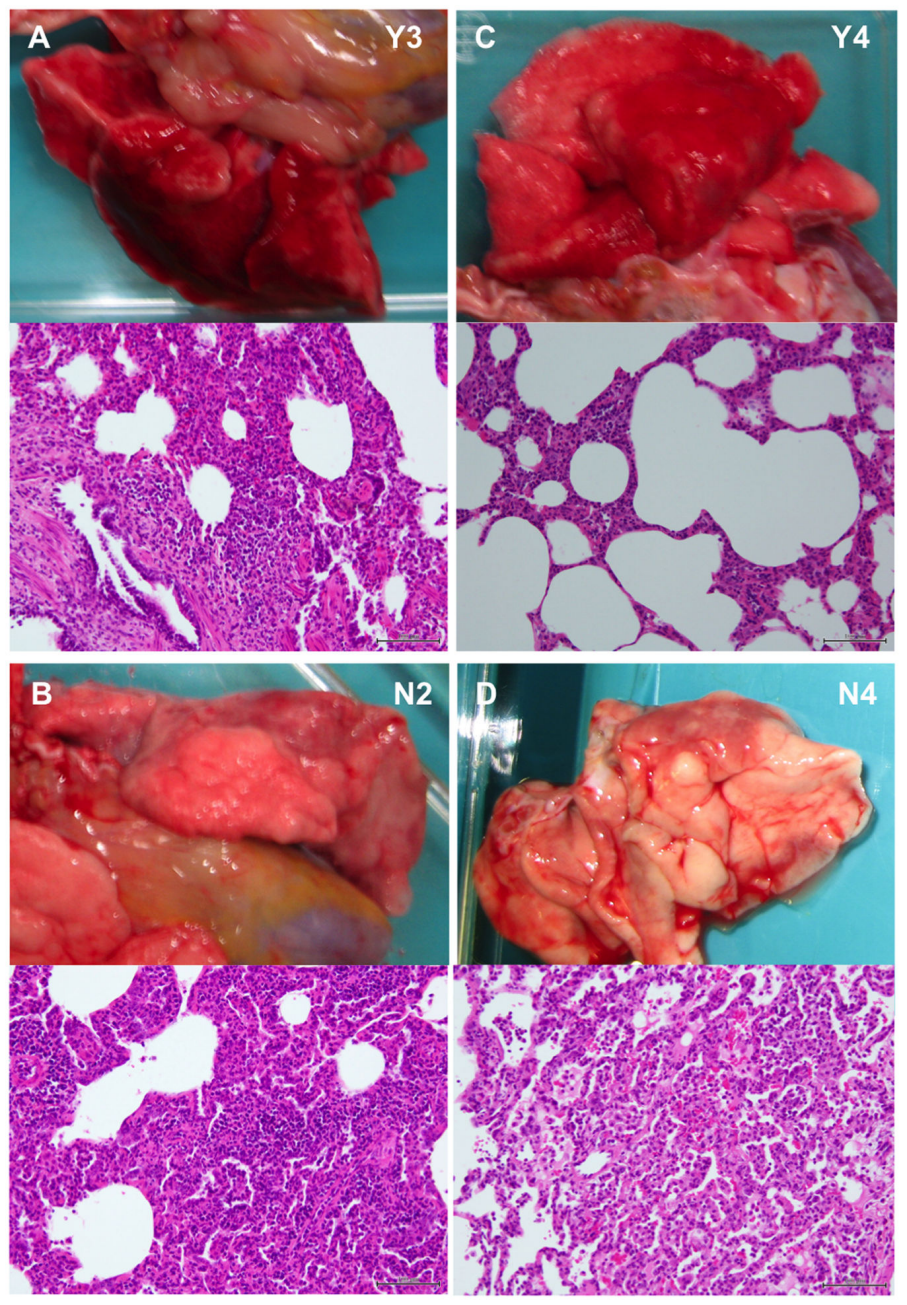
Histological analysis of viral pneumonia in immunosuppressed macaques. Macaques were autopsied 7 days after virus infection. Lung tissues were stained with hematoxylin and eosin (H & E) and representative figures are shown. (A) A macaque, Y3, infected with YOK91 without immunosuppression, (B) A macaque, N2, infected with NRT1 without immunosuppression, (C) A macaque, Y4, infected with YOK91 with immunosuppression, (D) A macaque, N4, infected with NRT1 with immunosuppression. Bars in microscopic photos indicate 100 µm.

We histologically examined lung tissues 7 days after infection. Without immunosuppression, congestion in lungs of the macaques infected with NRT1 and YOK91 was macroscopically observed (Figure 6A, B). Microscopically, both NRT1 and YOK91 induced severe lymphoid infiltration in the septum and alveolar space, resulting in thickening of alveolar walls (Figure 6A, B), and air content in the alveoli was decreased, especially in the lung of the immunocompetent macaque infected with NRT1 (Figure 6B). These results were compatible with alveolar and interstitial pneumonia. In the immunosuppressed macaques infected with YOK91 or NRT1, mild and local congestion in the lung was macroscopically observed (Figure 6C, D). Microscopically, lymphocyte infiltration into the septum and alveoli of the lung of the macaque infected with YOK91 was minimal (Figure 6C). In the lung of the macaque infected with NRT1, a small number of lymphocytes and macrophages infiltrated into alveoli but not in the septum (Figure 6D). Therefore, lymphocyte and leukocyte responses in the immunosuppressed macaques infected with YOK91 or NRT1 were less severe than those in the immunocompetent macaques, but inflammatory cytokine responses in lungs of the immunosuppressed macaques were more vigorous than those in the immunocompetent macaques (Figure 5).

## Discussion

In the present study, we revealed that the pandemic (H1N1) 2009 virus and the seasonal Russian flu virus propagated in immunosuppressed macaques and that inflammatory cytokines and chemokines were produced in lungs of macaques treated with CP and CA. The Russian flu virus was detected not only in the upper airway but also in the lower airway under the immunosuppressed condition. The pandemic (H1N1) 2009 virus was detected in both the upper and lower airways without immunosuppression, and higher virus titers in the lungs were detected in immunosuppressed macaques than in immunocompetent macaques. In addition, the pandemic (H1N1) 2009 virus was detected in non-respiratory organs of immunosuppressed macaques. These results indicated that a high level of virus propagation inducing cytokine production was related to severe morbidity in immunosuppressed macaques, that immunosuppression allowed the virus to spread to organs other than the main propagation sites in immunocompetent macaques and that non-highly pathogenic influenza virus systemically replicated under the immunocompromised condition.

In immunosuppressed macaques infected with either virus, infiltration of leukocytes was minimal in the lungs and congestive edema was observed in alveoli probably due to cytokines and chemokines. Therefore, we speculated that virus in the lungs of immunosuppressed macaques might induce inflammatory cytokine/chemokine production by non-leukocytes, i.e., alveolar epithelial cells or stromal cells [28-31], though we could not identify cytokine-positive cells by our immunohistochemical staining (data not shown). Furthermore, if the pandemic (H1N1) 2009 virus was inhaled into the trachea and alveoli in immunocompromised hosts, the virus would propagate widely in the lungs and possibly other organs with higher virus titers than would seasonal Russian flu virus and it would induce a high level of IL-6 production, which causes morbidity [32]. As association of IL-6 and IL-1β levels with fever was shown in previous studies [33,34], the high temperature in immunosuppressed macaques, especially in the N4 macaque showing prolonged high body temperature (Figure S2), was thought to be related to the relatively high level of inflammatory cytokine production. In addition, weak TGF-β responses in the pandemic (H1N1) 2009 virus infection might enhance effects of inflammatory cytokines [35,36]. Therefore, it was thought that pathogenicity of the pandemic (H1N1) 2009 virus was higher than that of the seasonal Russian flu virus under the immunocompromised condition as well as immunocompetent condition.

Although virus titers in nasal samples of the immunosuppressed macaques on day 7 after pandemic (H1N1) 2009 virus inoculation were significantly higher than those in nasal samples of the immunocompetent macaques, the virus titer AUCs in the trachea and bronchus of macaques infected with the pandemic virus under the immunosuppressive condition were lower than those in the trachea and bronchus of macaques without immunosuppression as shown in Figure 3. Lower virus titers in the trachea and bronchus of immunosuppressed macaques might be caused by the inhibitory effect of CA on influenza virus replication [37]. However, no difference in virus titer AUCs in nasal swab samples of macaques infected with the pandemic virus was found, and the pandemic virus was detected in more tissues of the immunosuppressed macaques than in tissues of the immunocompetent macaques (Figure 4B). In mouse models infected with H3N2 and WSN virus, virus titers in lungs of mice treated with CA as well as those in lungs of mice treated with CP alone were higher than those in lungs of mice without CA treatment [38-40]. Furthermore, the concentrations of CA in plasma of macaques (Y4-Y6 and N4-N6) on day 0 (blood was collected before infection on the day of infection) were lower than 20 ng/ml (the detection limit), which was lower than the concentration showing inhibition of virus propagation in vitro [37]. Taken together, the results indicate that the effect of CA on viral propagation was minimal at the concentration in the present study and CA did not decrease morbidity caused by the pandemic (H1N1) 2009 influenza.

CA and CP are widely used for prevention of rejection in organ transplantations and treatments for cancers [41]. In patients under the treatment with the immunosuppressive drugs, pneumonia and mortality was reported more frequently than in non-immunosuppressed individuals during the pandemic in 2009 [42,43]. Furthermore, antibody responses by influenza virus vaccination in breast cancer patients treated with CP [44] and renal and lung transplant patients treated with CA [45,46] were impaired. In liver transplant patients treated with CA [47], T cell responses against vaccine antigens were impaired as shown in the present study. Therefore, our immunosuppressive macaques model would be used for extrapolation of pathogenicity and development of effective vaccination in immunocompromised patients.

Based on these features, the immunosuppressed macaque model would be also useful for evaluating the emergence of drug-resistant strains during treatment, such strains having been reported in immunocompromised patients [48,49]. Since acquired immune responses including antibody responses should be weak in immunocompromised hosts, anti-viral drugs might become a predominant selective pressure under an immunosuppressed condition. Therefore, if drug-resistant strains with mutations were detected in the immunosuppressed macaques treated with the anti-viral drug, we could focus surveillance using samples isolated from humans on the predicted positions in viral proteins of drug-resistant strains.

Inflammatory responses observed in immunosuppressed macaques infected with the virus appeared to be contradictory to the immunocompromised condition. It has been reported that lipopolysaccharide (LPS)-induced fever was independent of prostaglandin E_2,_ suggesting that fever dependent on toll-like receptor (TLR) signals, e.g. TLR7, having been shown to be a receptor for virus RNA [50], might not be inhibited by cyclooxygenase (COX)-2 inhibitors or non-steroid anti-inflammatory drugs [51]. Furthermore, corticosteroid therapy in patients with pandemic (H1N1) 2009 virus infection was shown to be associated with higher mortality than that without steroid treatment [52], suggesting that corticosteroid therapy might be hazardous as shown in mice infected with H5N1 highly pathogenic avian influenza virus [53]. These results suggest that anti-inflammatory drugs broadly used in humans do not necessarily suppress inflammation and the cytokine storm (hypercytokinemia) [54]. Therefore, development of other types of anti-hypercytokinemia treatment rather than anti-inflammatory drugs is required for immunocompromised patients with influenza virus infection and for patients with highly pathogenic avian influenza virus infection [55,56]. We will analyze the pathogenicity of highly pathogenic avian influenza virus and anti-hypercytokinemia treatment in immunocompromised macaques in the future.

## Materials and Methods

### Ethics Statement

This study was carried out in strict accordance with the Guidelines for the Husbandry and Management of Laboratory Animals of the Research Center for Animal Life Science at Shiga University of Medical Science and Standards Relating to the Care and Management, etc. of Experimental Animals (Notification No. 6, March 27, 1980 of the Prime Minister’s Office, Japan). The protocol was approved by the Shiga University of Medical Science Animal Experiment Committee (Permit numbers: 2009-8-6H and 2010-10-1). The Research Center for Animal Life Science at Shiga University of Medical Science has a permit for importation of cynomolgus macaques. Regular veterinary care and monitoring, balanced nutrition and environmental enrichment were provided by the Research Center for Animal Life Science at Shiga University of Medical Science. The macaques were euthanized at endpoint on 7 days after virus inoculation using ketamine/xylazine followed by intravenous injection of pentobarbital (200 mg/kg). Animals were monitored every day during the study to be clinically scored as described in Table S1 [57] and to undergo veterinary examinations to help alleviate suffering. Animals would be euthanized if their clinical scores reached 15 (a humane endpoint) though no animals showed symptoms scored as 15 in the present study.

### Animals

Five- to seven-year-old female cynomolgus macaques from Vietnam and the Philippines (Ina Research Inc., Ina, Japan) were used. The cynomolgus macaques used in the present study were healthy young adults. All procedures were performed under ketamine and xylazine anesthesia, and all efforts were made to minimize suffering. Food pellets of CMK-2 (CLEA Japan, Inc., Tokyo, Japan) were provided once a day after recovery from anesthesia and drinking water was available *ad libitum*. Animals were singly housed in the cages equipping bars to climb up and puzzle feeders for environmental enrichment under controlled conditions of humidity (40 ± 5%), temperature (25 ± 1°C), and light (12-h light/12-h dark cycle, lights on at 8: 00 A.M.). In the text and figures, individual macaques are distinguished by the following abbreviations: Y1, Y2, Y3 for macaques inoculated with YOK91 without immunosuppression, Y4, Y5, Y6 for macaques inoculated with YOK91 with immunosuppression, N1, N2, N3 for macaques inoculated with NRT1 without immunosuppression, and N4, N5, N6 for macaques inoculated with NRT1 with immunosuppression. The absence of influenza A virus nucleoprotein-specific antibodies in their sera was confirmed before experiments using an antigen-specific enzyme-linked immunosorbent assay (ELISA), AniGen AIV Ab ELISA (Animal Genetics Inc., Kyonggi-do, Korea), for currently circulating influenza virus. Two weeks before virus inoculation, a telemetry probe (TA10CTA-D70, Data Sciences International, St. Paul, MN) was implanted in the peritoneal cavity of each macaque under ketamine/xylazine anesthesia followed by isoflurane inhalation to monitor body temperature. Antibiotics and analgesics were used after the surgery. The macaques used in this study were free from B virus, hepatitis E virus, *Mycobacterium tuberculosis*, *Shigella* *spp.* , *Salmonella spp.*, and *Entamoeba histolytica*. All experiments using viruses were performed in the biosafety level 3 facility of the Research Center for Animal Life Science, Shiga University of Medical Science. Virus (3 × 10^6^TCID_50_/7ml) was inoculated into nostrils (0.5 ml for each nostril), oral cavity (0.5 ml for the surface of each tonsil), and trachea (5 ml). Under ketamine/xylazine anesthesia, 2 cotton sticks (TE8201, Eiken Chemical, Ltd., Tokyo, Japan) were used to collect fluid samples in nasal cavities and tracheas, and the sticks were subsequently immersed in 1 ml of phosphate-buffered saline (PBS) containing 0.1% bovine serum albumin (BSA) and antibiotics. A bronchoscope (MEV-2560, Machida Endoscope Co. Ltd., Tokyo, Japan) and cytology brushes (BC-203D-2006, Olympus Co., Tokyo, Japan) were used to obtain bronchial samples. The brushes were immersed in 1 ml PBS with BSA.

### Viruses

The seasonal influenza virus A/Yokohama/91/2007 (H1N1) (kindly provided by Dr. Chiharu Kawakami, Yokohama City Institute. National Center for Biotechnology Information (NCBI) taxonomy database ID: 568510) was propagated in Madin-Darby canine kidney (MDCK) cells twice at Yokohama City Institute and once in MDCK cells at Shiga University of Medical Science [58]. The pandemic influenza virus A/Narita/1/2009 (H1N1) pdm (kindly provided by Dr. Takato Odagiri, National Institute of Infectious Disease (NIID), Japan. NCBI taxonomy database ID: 645520) [59,60] was cultured twice in embryonated eggs in NIID and once in MDCK cells at Shiga University of Medical Sciences.

In order to assess virus replication, serial dilutions of swab samples and homogenized tissue samples were inoculated onto confluent MDCK cells as described previously [61]. Cytopathic effects were examined under a microscope 72 h later.

### Immunosuppressive treatment

Cyclophosphamide (Nakalai Tesque, Kyoto, Japan) was dissolved in saline (Otsuka Pharmaceutical Co., Ltd., Tokushima, Japan) at 40 mg/ml, stored at 4 °C after filtration, and used within 2 days of preparation. CP was intravenously administered by bolus injection. Cyclosporine A (Novartis Pharma, Basel, Switzerland, 100 mg/ml) was orally administered by catheters. The concentration of CA in plasma was measured by SRL Inc., Tokyo, Japan, with a double-antibody radioimmunoassay method.

### Blood cells

The numbers of leukocytes in blood were counted using a hemocytometer and a microscope. For flow cytometry, whole blood cells were stained with phycoerythrin (PE)-conjugated anti-CD8 (clone: SK1, eBioscience, San Diego, CA), allophycocyanin (APC)-conjugated anti-CD4 (clone: OKT-4, eBioscience), FITC-conjugated anti-CD14 (clone: M5B2, BioLegend, Inc., San Diego, CA), and PE-conjugated anti-CD20 (clone: 2H7, BioLegend). Dead cells were excluded by staining with ethidium monoacetate (EMA, Molecular Probes, Inc., Eugene, OR). Red blood cells were lysed with tris-ammonium chloride. Cells were fixed with PBS containing 4% paraformaldehyde before flow cytometer analysis.

### Cytokine assay

In the experiment for which results are shown in Figure 1, white blood cells were purified from 2 ml of peripheral blood using a density gradient (Wako Pure Chemical Industries Ltd., Osaka, Japan). After washing twice, 1 × 10^5^ cells were cultured with anti-CD3 (clone: SP34), anti-CD28 (clone: CD28.2) and anti-CD49d (clone: 9F10) antibodies (0.5 µg/ml, eBioscience) or with phorbol 12-myristate 13-acetate (PMA) (0.1 µg/ml, Nacalai Tesque) and ionomycin (1 µg/ml, Nacalai Tesque) in 96-well U-bottom plates for 24 h and supernatants were collected. The concentrations of IL-2 in culture supernatants were determined using MAX Deluxe SET Human IL-2 (BioLegend).

In the experiment for which results are shown in Figure 5, lung tissues obtained at autopsy on day 7 after the challenge infection were used after homogenization. Tissue homogenates were prepared as 10% w/v solution. The concentrations of IFN-β and total TGF-β1 were measured using the VeriKine^TM^ Human Interferon Beta ELISA kit (Pistka Biomedical Laboratories, Inc., PBL Interferon Source, Piscataway, NJ) and the human TGF-β1 ELISA kit (R&D Systems, Inc., Minneapolis, MN), respectively. The concentrations of TNF-α, IL-1β, IL-6, IL-18, and MCP-1 in homogenates were measured using the Milliplex MAP non-human primate cytokine panel and Luminex200 (Millipore Corp., Billerica, MA).

### Histological examination

After autopsy, lung tissue samples were fixed with 10% formalin, and embedded in paraffin. Sections were stained with hematoxylin and eosin (H&E). Influenza virus nucleoprotein (NP) was stained with anti-NP antibody, HB65, after treatment of sections with pronase [62]. After incubation with anti-mouse immunoglobulin antibody conjugated with horseradish peroxidase, NP was detected with diaminobentidin (Nichirei Bioscience Inc., Tokyo, Japan).

## Supporting Information

Figure S1 **Flow cytometric analysis of peripheral blood cells in immunosuppressed macaques.** Cynomolgus macaques were administered CP and CA as indicated in Table 1. Figure S1 A and B show results of the low and high dose regimens, respectively. Blood was collected on the indicated days after immunosuppression. Top row: dot plots of FSC and SSC. Blue: R1 (low FSC/low SSC, lymphocytes), orange: R2 (high FSC/low SSC, mainly monocytes), red: R3 (high FSC/high SSC, granulocytes). Second row: dot plots of CD4 and CD8 gated on R1 cells. Third row: dot plots of CD14 and CD20 gated on R1. Bottom row: dot plots of CD14 and CD20 gated on R2.
(TIF)

Figure S2 **Body temperature after immunosuppression and virus infection.** Cynomolgus macaques were administered a high dose of CP and CA as shown in Table 2 (blue lines, immunosuppression). Control macaques were administered saline (red lines, immunocompetent). Seasonal influenza virus A/Yokohama/91/2007 (H1N1) (left, YOK91) or pandemic influenza virus A/Narita/1/2009 (H1N1) (right, NRT1) was inoculated into the nostrils, oral cavity, and trachea of macaques on day 0 (red arrowheads). Body temperature of macaques was recorded using telemetry transmitters and a computer. Body temperature of one macaque, N6, with immunosuppression and infection with NRT1 was not recorded due to battery power shortage.
(TIF)

Figure S3 **Blood cell populations in immunosuppressed macaques infected with influenza virus.** Macaques were administered CP and CA from day -7 to day 0 and then inoculated with influenza virus YOK91, Y1-Y6, or NRT1, N1-N6, on day 0. Blood was collected on the indicated days. Blood cells were stained with fluorescence-conjugated antibodies specific for CD4, CD8, CD14, and CD20. The concentration of each population was calculated using white blood cell counts shown in Figure 2 and the percentage determined by flow cytometric analysis. The concentrations of CD4^+^, CD8^+^, and CD20^+^ cells were calculated in R1 (low FSC/low SSC) as shown in Figure S1. The concentrations of CD14^+^ cells were calculated in R2 (high FSC/low SSC). The concentrations of granulocytes were calculated in R3 (high FSC/high SSC). The left y-axis indicates numbers of CD4^+^, CD8^+^, CD14^+^, and CD20^+^ cells. The right y-axis indicates numbers of granulocytes.
(TIF)

Figure S4 **Immunohistochemical staining for influenza virus NP in organs of immunocompetent and immunocompromised macaques.** Tissues were collected as described in the legend for Figure 4 and stained with anti-influenza virus NP antibody. (A) Lung of a control macaque without immunosuppression and virus infection. No inflammation and NP-positive cells were observed. (B) Lung of a macaque infected with YOK91 without immunosuppression (Y3). (C) Lung of a macaque infected with YOK91 with immunosuppression (Y4). (D) Bronchiole in the lung of a macaque infected with NRT1 without immunosuppression (N1). (E) Lung, (F) cerebellum, (G) descending colon, (H) urinary bladder of a macaque infected with NRT1 with immunosuppression. Bars in microscopic photos indicate 50 µm.
(TIF)

Table S1 **Clinical scoring used in this study.** Animals were monitored every day during the study to be clinically scored. Animals would be euthanized if their clinical scores reached 15 (a humane endpoint).
(DOCX)
