# Supplementary figures and images for: Pathogenicity of Pandemic H1N1 Influenza A Virus in Immunocompromised Cynomolgus Macaques
Source: PLoS One. 2013 Sep 23;8(9):e75910. doi: 10.1371/journal.pone.0075910 (PMC3781065; doi:10.1371/journal.pone.0075910)

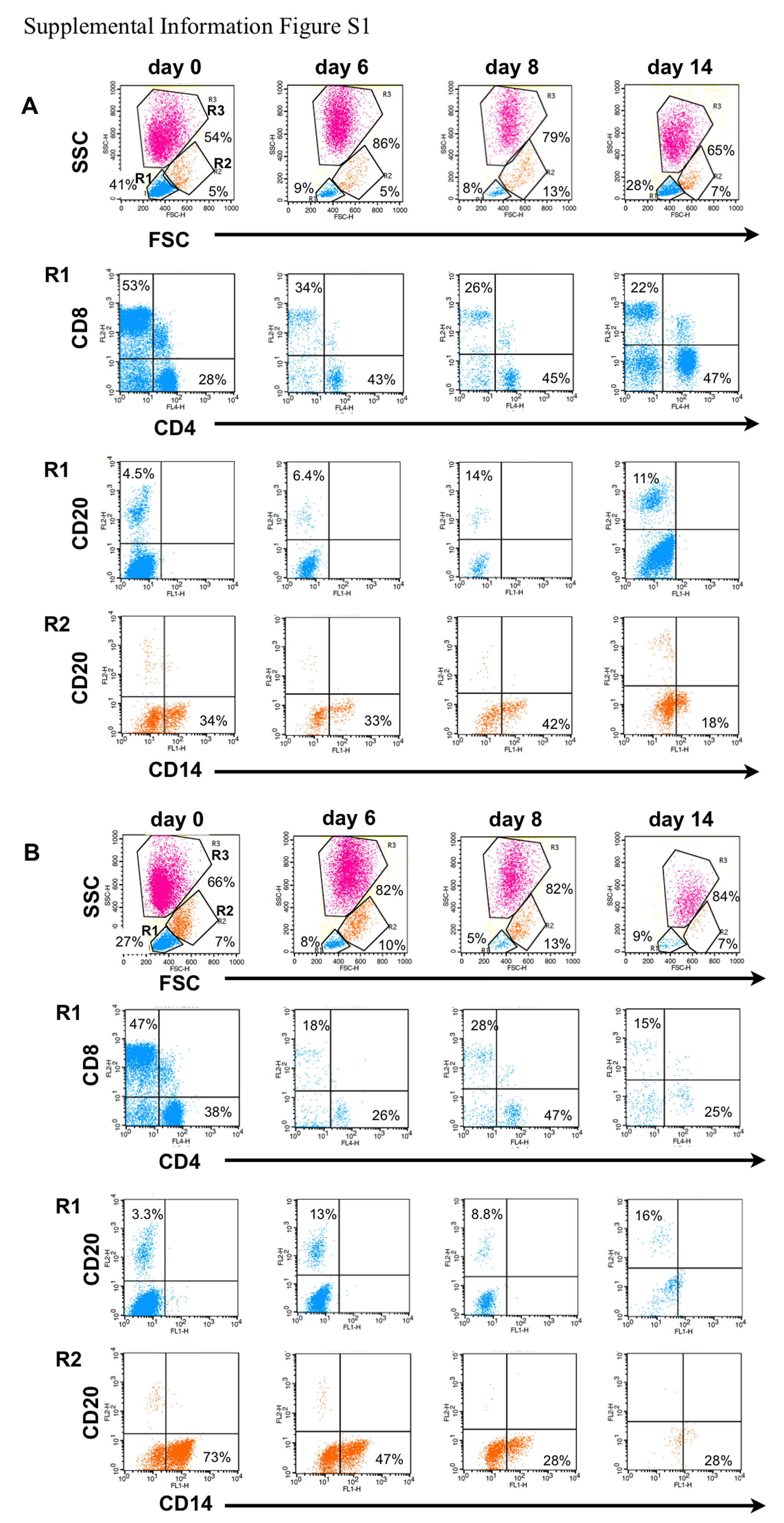

Supplement: Figure S1 — Flow cytometric analysis of peripheral blood cells in immunosuppressed macaques. Cynomolgus macaques were administered CP and CA as indicated in Table 1. Figure S1 A and B show results of the low and high dose regimens, respectively. Blood was collected on the indicated days after immunosuppression. Top row: dot plots of FSC and SSC. Blue: R1 (low FSC/low SSC, lymphocytes), orange: R2 (high FSC/low SSC, mainly monocytes), red: R3 (high FSC/high SSC, granulocytes). Second row: dot plots of CD4 and CD8 gated on R1 cells. Third row: dot plots of CD14 and CD20 gated on R1. Bottom row: dot plots of CD14 and CD20 gated on R2. (TIF) [file pone.0075910.s001.tif]

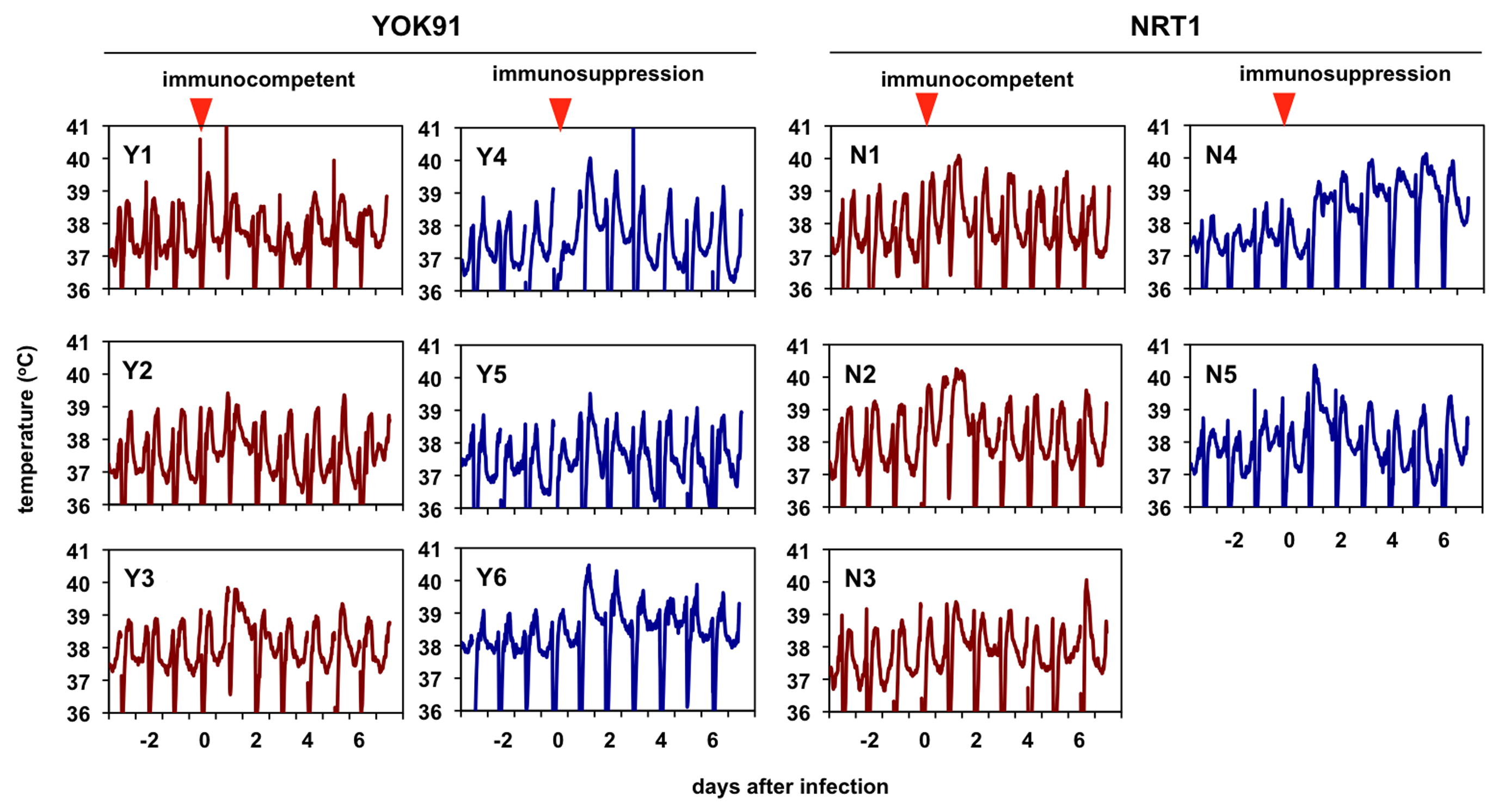

Supplement: Figure S2 — Body temperature after immunosuppression and virus infection. Cynomolgus macaques were administered a high dose of CP and CA as shown in Table 2 (blue lines, immunosuppression). Control macaques were administered saline (red lines, immunocompetent). Seasonal influenza virus A/Yokohama/91/2007 (H1N1) (left, YOK91) or pandemic influenza virus A/Narita/1/2009 (H1N1) (right, NRT1) was inoculated into the nostrils, oral cavity, and trachea of macaques on day 0 (red arrowheads). Body temperature of macaques was recorded using telemetry transmitters and a computer. Body temperature of one macaque, N6, with immunosuppression and infection with NRT1 was not recorded due to battery power shortage. (TIF) [file pone.0075910.s002.tif]

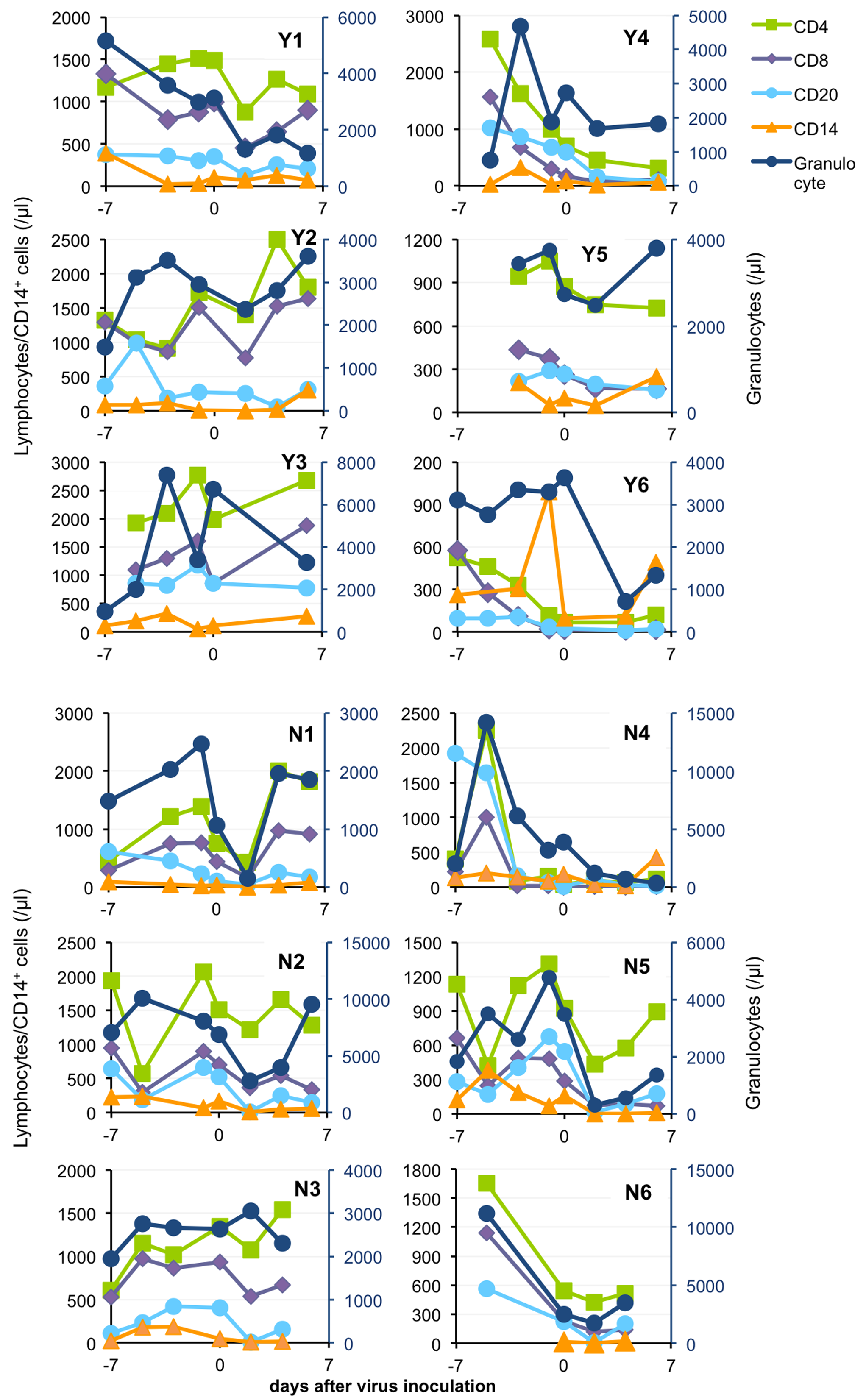

Supplement: Figure S3 — Blood cell populations in immunosuppressed macaques infected with influenza virus. Macaques were administered CP and CA from day -7 to day 0 and then inoculated with influenza virus YOK91, Y1-Y6, or NRT1, N1-N6, on day 0. Blood was collected on the indicated days. Blood cells were stained with fluorescence-conjugated antibodies specific for CD4, CD8, CD14, and CD20. The concentration of each population was calculated using white blood cell counts shown in Figure 2 and the percentage determined by flow cytometric analysis. The concentrations of CD4+, CD8+, and CD20+ cells were calculated in R1 (low FSC/low SSC) as shown in Figure S1. The concentrations of CD14+ cells were calculated in R2 (high FSC/low SSC). The concentrations of granulocytes were calculated in R3 (high FSC/high SSC). The left y-axis indicates numbers of CD4+, CD8+, CD14+, and CD20+ cells. The right y-axis indicates numbers of granulocytes. (TIF) [file pone.0075910.s003.tif]

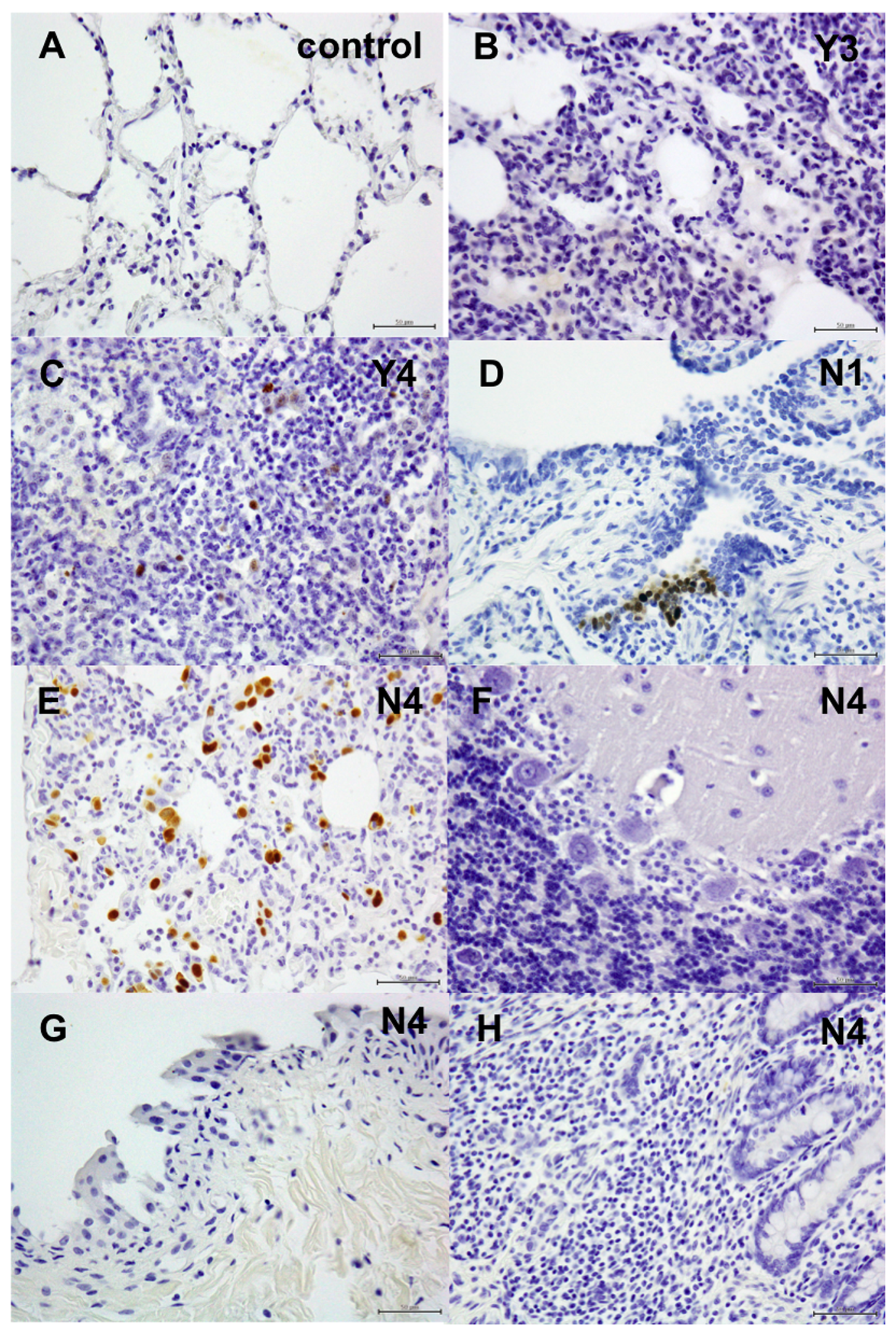

Supplement: Figure S4 — Immunohistochemical staining for influenza virus NP in organs of immunocompetent and immunocompromised macaques. Tissues were collected as described in the legend for Figure 4 and stained with anti-influenza virus NP antibody. (A) Lung of a control macaque without immunosuppression and virus infection. No inflammation and NP-positive cells were observed. (B) Lung of a macaque infected with YOK91 without immunosuppression (Y3). (C) Lung of a macaque infected with YOK91 with immunosuppression (Y4). (D) Bronchiole in the lung of a macaque infected with NRT1 without immunosuppression (N1). (E) Lung, (F) cerebellum, (G) descending colon, (H) urinary bladder of a macaque infected with NRT1 with immunosuppression. Bars in microscopic photos indicate 50 µm. (TIF) [file pone.0075910.s004.tif]
