# Supplementary material for: Pathogenicity of Pandemic H1N1 Influenza A Virus in Immunocompromised Cynomolgus Macaques
Source: PLoS One. 2013 Sep 23;8(9):e75910. doi: 10.1371/journal.pone.0075910 (PMC3781065; doi:10.1371/journal.pone.0075910)
Supplement: Table S1 — Clinical scoring used in this study. Animals were monitored every day during the study to be clinically scored. Animals would be euthanized if their clinical scores reached 15 (a humane endpoint). (DOCX) [file pone.0075910.s005.docx]

**Supporting Information**

**Table S1. Clinical scoring used in this study**

| **Parameter** | **Degree of parameter** | **Possible score** |
| --- | --- | --- |
| Fever | Normal (< 39 ^o^C) | 0 |
|  | Elevated temperature (39-40 ^o^C) | 3 |
|  | High temperature (> 40 ^o^C) | 5 |
| Posture | Piloerection of body hair | 1 |
|  | Decreased activity, decreasing normal behavior/Occasionally lies down, huddled, active when people in room | 2 |
|  | Huddled on camera, active when people in room/Lie down, will get up when approached, use cage for support | 3 |
|  | Huddled when people in room, shaking, toes and hands clenched/Lie down, will not get up when approached or prompted | 5 |
| Respiration | Increased or Decreased; mild cough and clear nasal discharge | 3 |
|  | Labored breathing through mouth; severe cough and severe nasal discharge | 5 |
| Appetite | Slightly decreased | 1 |
|  | Decreased | 2 |
|  | Severely decreased | 5 |
| Skin | Flushed appearance | 2 |
|  | Visible rash | 2 |
|  | Bleeding | 5 |
